# Supplementary material for: A multi-scale microstructure to address the strength-ductility trade off in high strength steel for fusion reactors
Source: Nat Commun. 2025 Mar 20;16:2746. doi: 10.1038/s41467-025-58042-8 (PMC11926085; doi:10.1038/s41467-025-58042-8)
Supplement: Supplementary file 1 — Supplementary Information [file 41467_2025_58042_MOESM1_ESM.pdf]

Supplementary Materials for

A multi-scale microstructure to address the strength-ductility trade off in high strength steel for fusion reactors

Supplementary Table 1. Chemical compositions of the RAFM steels (wt. %)

Materials	C	Si	Mn	Cr	V	Ta	Ti	W	Fe
Sheffield RAFM steel	0.11	0.25	0.4	9	0.2	0.07	0.01	1.1	Bal.
Eurofer97 steel	0.11	--	0.55	8.95	0.2	0.12	--	1.06	Bal.

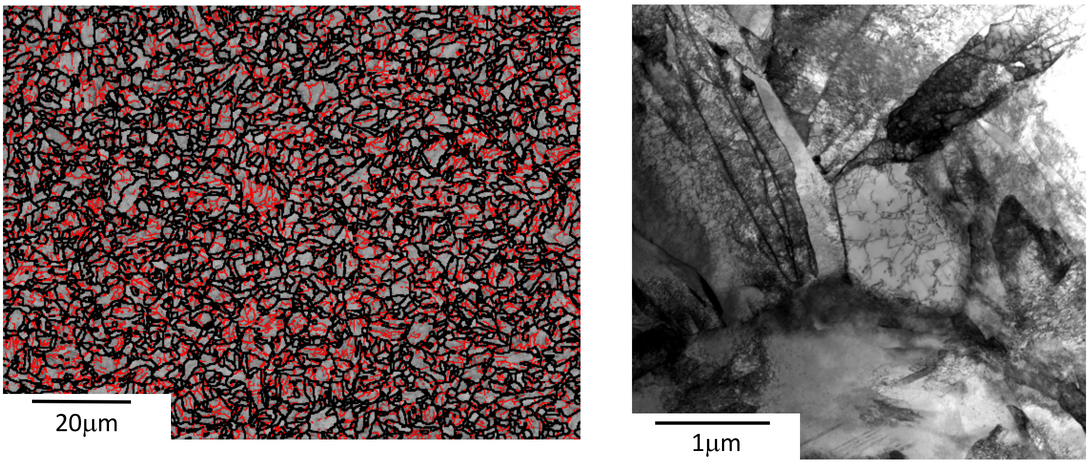

Supplementary Figure 1. Grain structure. a EBSD map showing the microstructure after normalization at 980 for 1h following water quenching. **b** TEM bright field image showing a fine ferrite gain (α_4 grain) surrounded by as-quenched martensite.

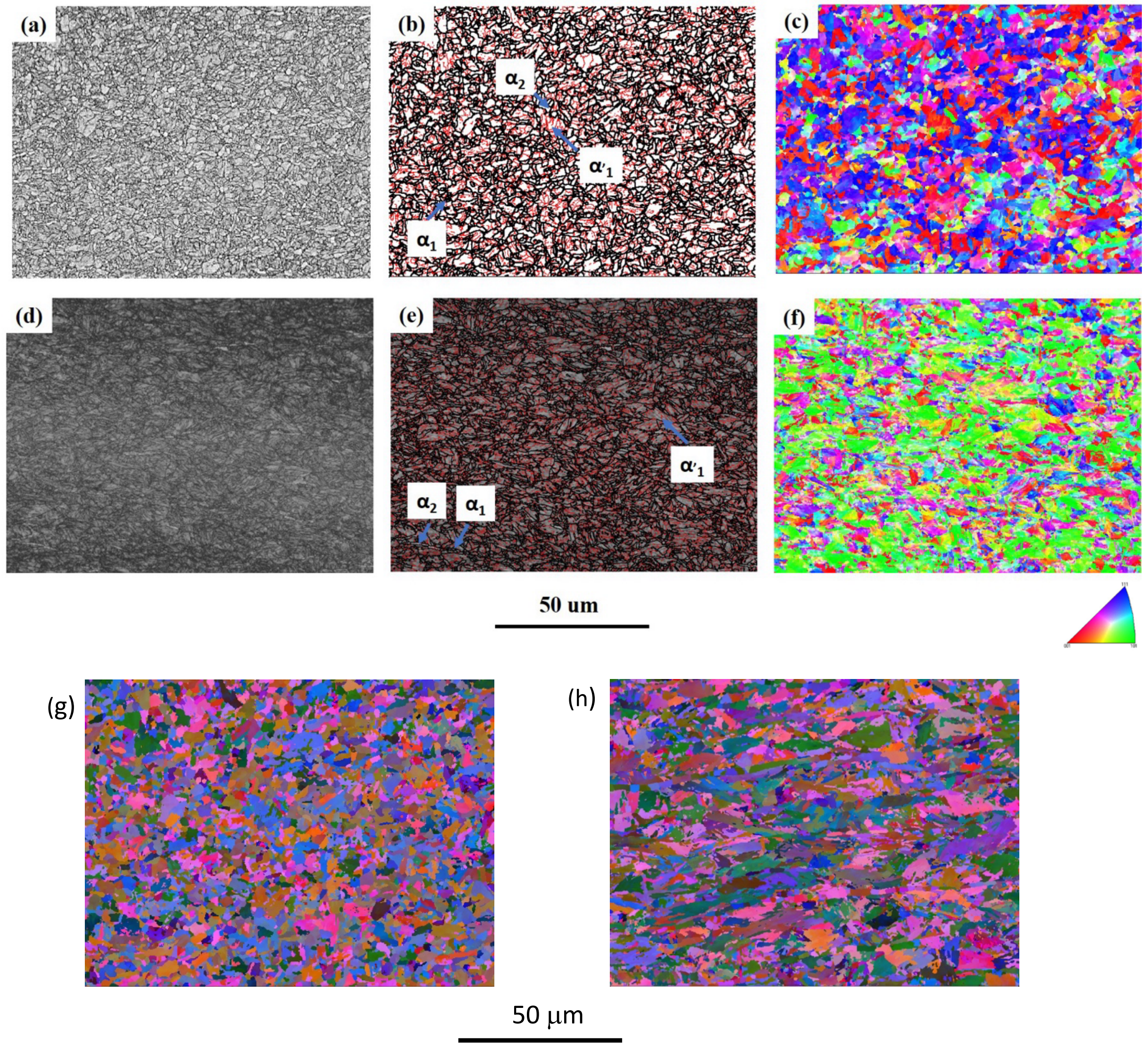

Supplementary Figure 2. EBSD showing grain structure. EBSD images giving a comparison of the Stage 2 (a,b,c,g) and Stage 3 (d,e,f,h) steels in the hot rolled condition, before heat treatment. **a** and **d** are band contrast images; **b** and **e** give grain boundary maps of the same area (black lines indicate HAGBs (>5˚) and red lines indicate LAGBs (<5˚)); **c** and **f** Inverse pole figure (IPF) maps of the same area. Note that while the Stage 2 shows equiaxed grains, after Stage 3 the grains are elongated, consistent with intercritical deformation. **g** and **h** Euler colour maps at slightly higher magnification, showing the generally equiaxed structure after Stage 2 **g** and the elongated structure after Stage 3 **h**.

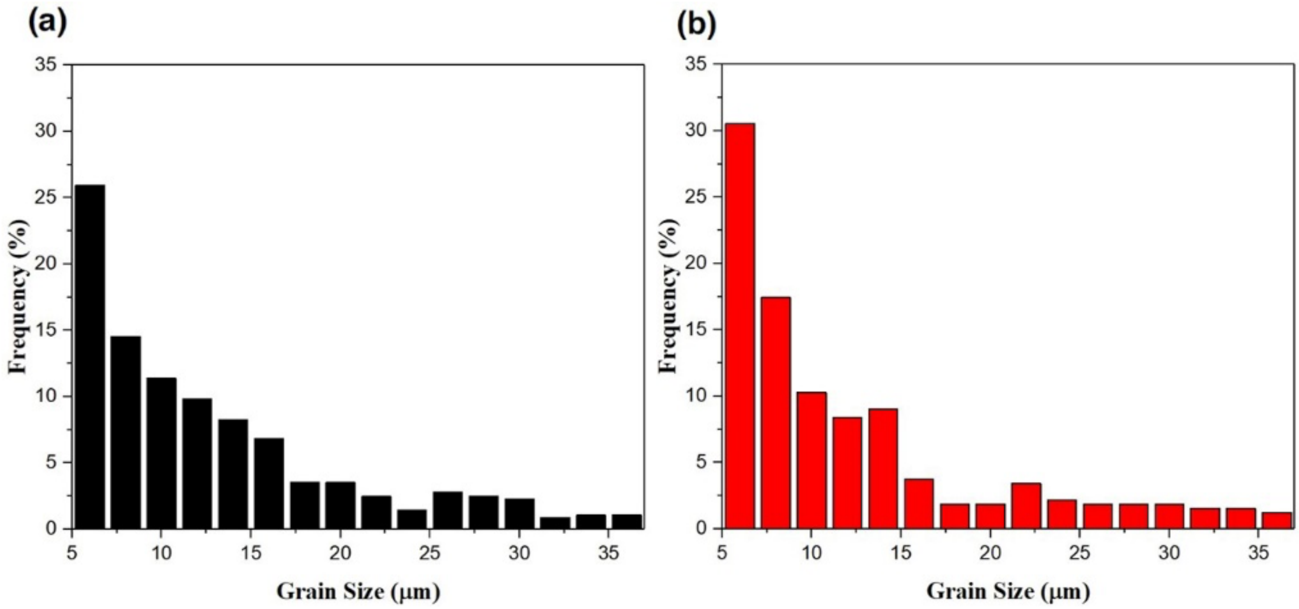

Supplementary Figure 3. Grain size distribution. Grain size distribution derived from EBSD maps from **a** Stage 2 RAFM steel; **b** Stage 3 RAFM steel. An average of 400 grains has been calculated.

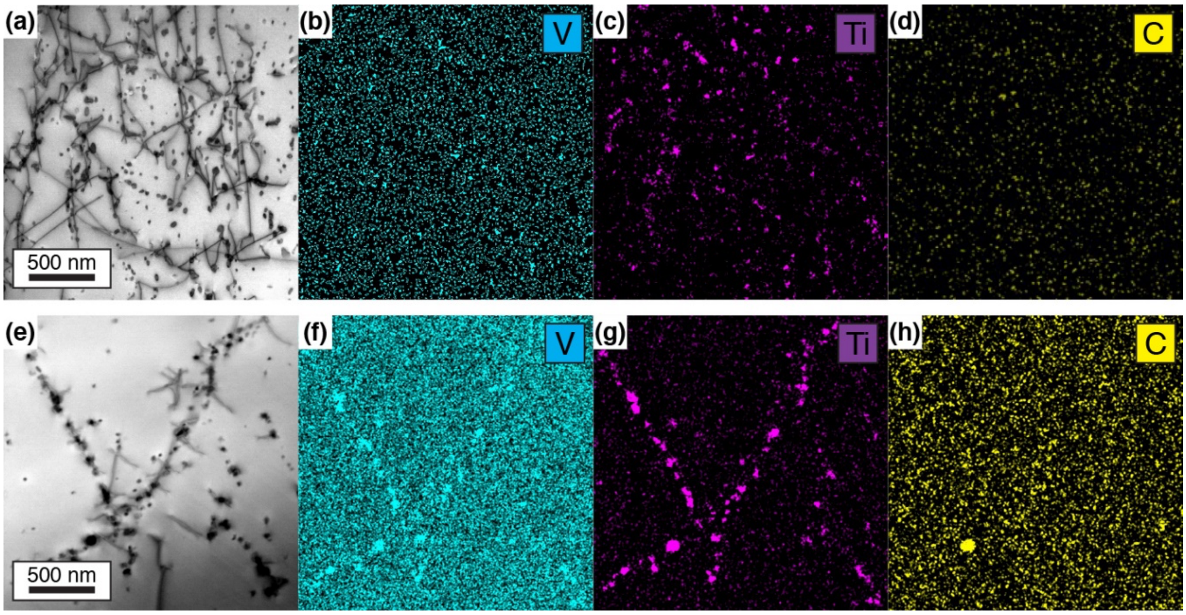

Supplementary Figure 4. Precipitate distributions. STEM-BF and EDS maps in the coarser ferrite region in the Stage 3 RAFM steel, showing the finer scale (V,Ti)C located on the dislocations and dislocation cells: **a** STEM-BF of nanoscale precipitates pinned on the dislocations; **b** V map from **a**; **c** Ti map from **a**; **d** C map from **a**; **e** STEM-BF of nanoscale precipitates pinned on the dislocation cell structures; **f** V map from **e**; **g** Ti map from **e**; **h** C map from **e**.

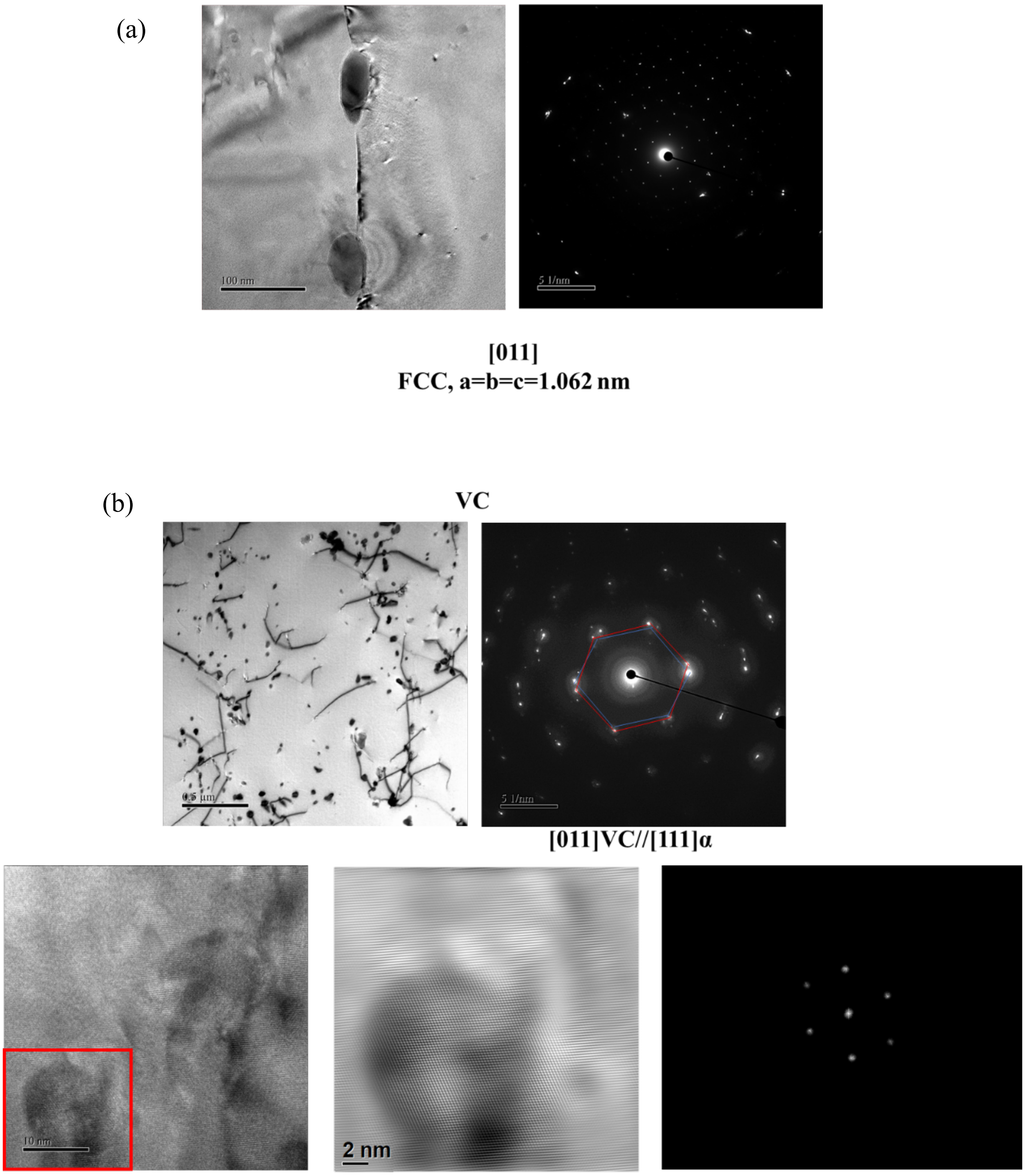

Supplementary Figure 5. Precipitate identification. a Bright field TEM and its corresponding selected area diffraction used to identify the $(Fe,Cr)_{23}C_6$. **b** Bright field TEM and associated selected area diffraction, and high resolution TEM with associated Fourier transform, identifying the (V,Ti)C carbides.

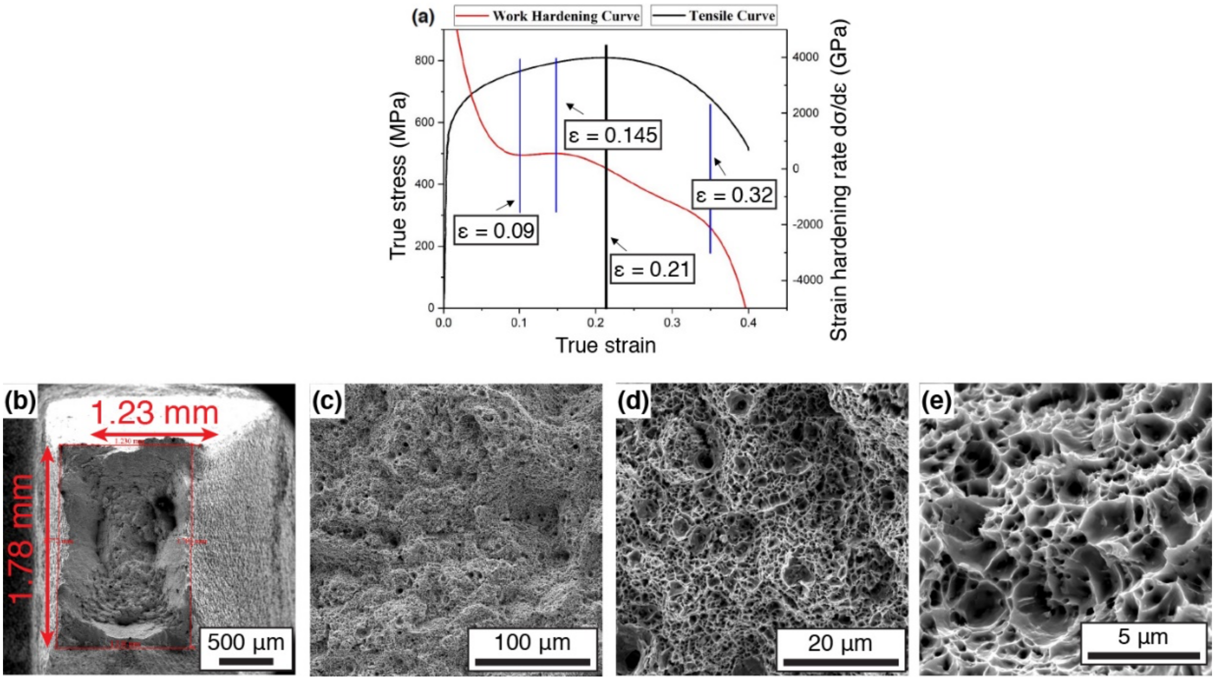

Supplementary Figure 6. Tensile work hardening and fracture surface appearance. a True stress-strain curve of the Stage 3 RAFM steel (curve taken as representative of 3 different repeat tests), and **b-e** SEM images of the fracture surface of specimens: **b** low magnification of fracture surface; **c-e** magnification increasing on the dimpled fracture surface.

Supplementary Table 2. Tensile values measured for the three steels.

Steel	Yield strength (MPa)	UTS (MPa)	Uniform elongation (%)	Total elongation (%)
Eurofer97		648	10.6	24
Stage 2	398	693	11.5	31
Stage 3	587	717	11.7	49

\

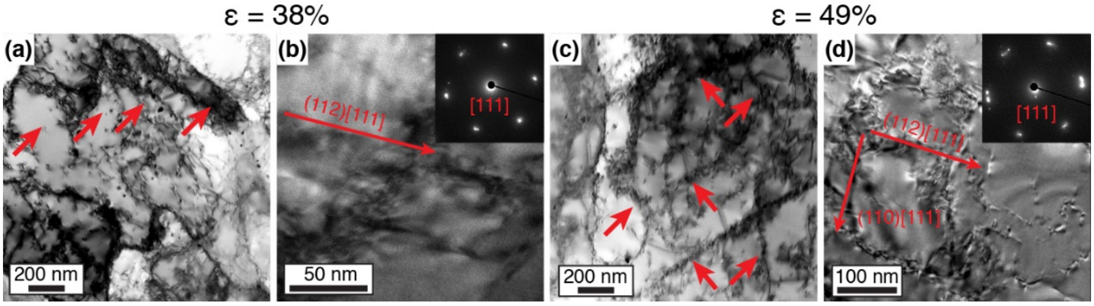

Supplementary Figure 7. Dislocation substructures. Bright field (S)TEM micrographs and the corresponding dark field images of the Stage 3 processed RAFM steel showing planar dislocation structures **a, b** tensile strain of 38% with **a** BF STEM image; **b** BF TEM image and corresponding selected area diffraction pattern (SADP) showing (112)[111] OR relationship; **c, d** tensile strain of 49%; **c** BF STEM image; **d** BF TEM image and corresponding selected area diffraction pattern (SADP) showing two types of (110)[111] and (112)[111] OR relationship.

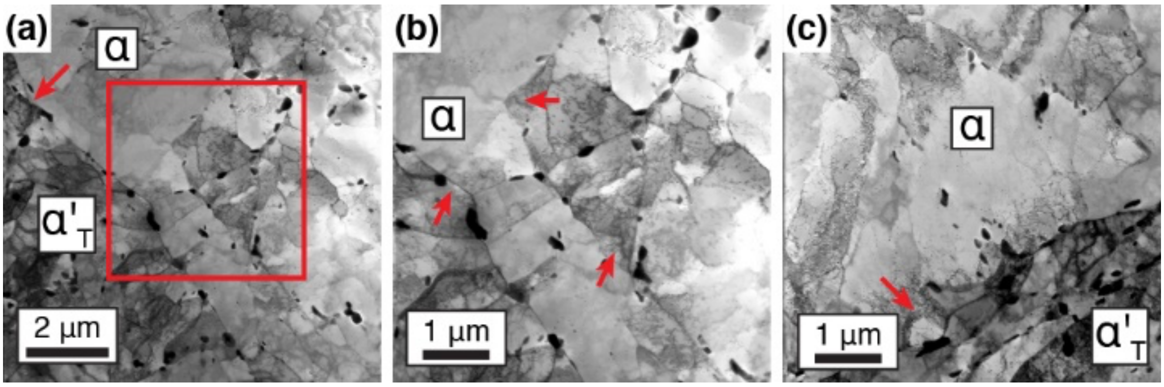

Supplementary Figure 8. Tensile strain microstructure. Bright field STEM micrographs of the Stage 3 RAFM steel at the tensile strain of 49%: **a** low magnification of BF STEM showing bulging dual phase boundaries, marked by red arrows; **b** high magnification of BF STEM from **a** in red rectangle; **c** high magnification of BF STEM from different region showing the high dislocation density near the dual phase boundaries.

Supplementary Table 3. Measured values of grain size, dislocation density, precipitate volume fraction and precipitate size.

Material	Grain size input (μm)	Dislocation density (10^{12} m^{-2})	Precipitate volume fraction (10^{-3})	Precipitate size (nm)
Stage 2	6.3	1.8	1.2	6
Stage 3	5.8	8.6	5.6	5.6

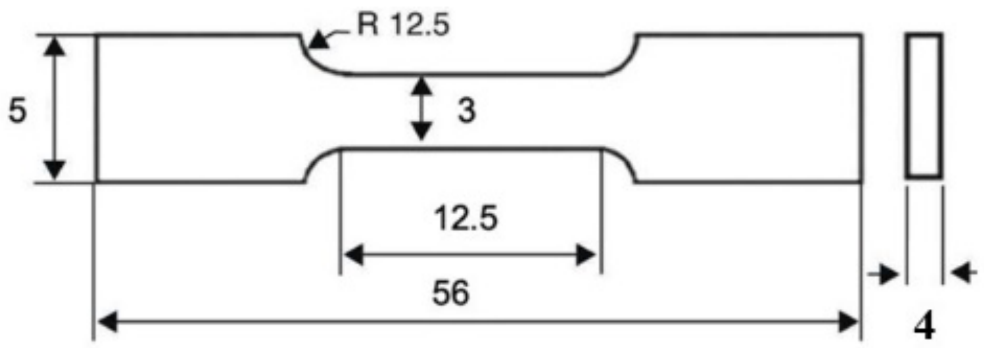

Supplementary Figure 9. Tensile sample geometry (lengths in mm)

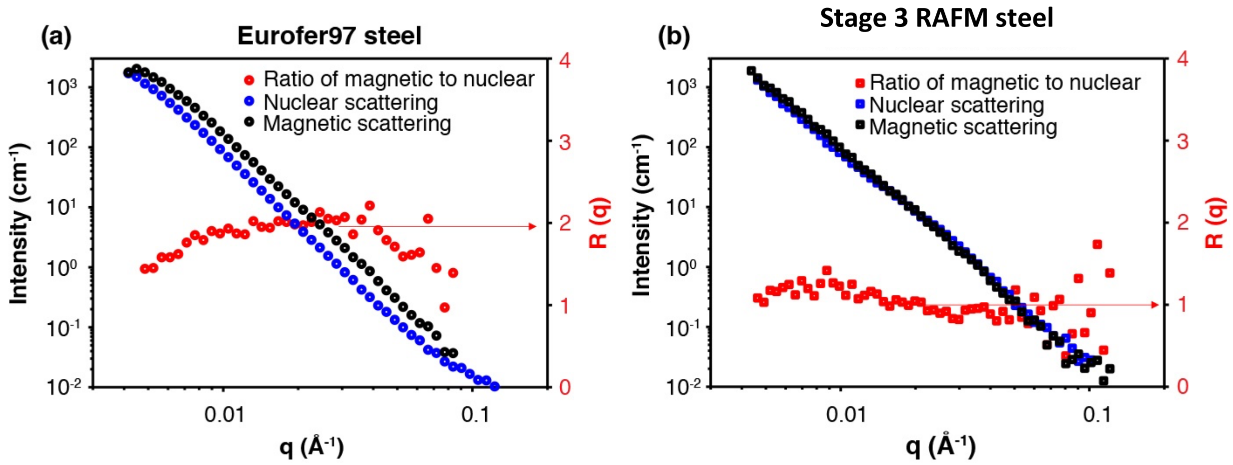

Supplementary Figure 10. Nuclear and magnetic small angle scattering data. One-dimensional nuclear and magnetic small angle neutron scattering patterns of I (left hand axis) and R(q) (right hand) axis versus scattering vector, q, obtained from **a** the Eurofer97 steel and **b** Stage 3 RAFM steel.

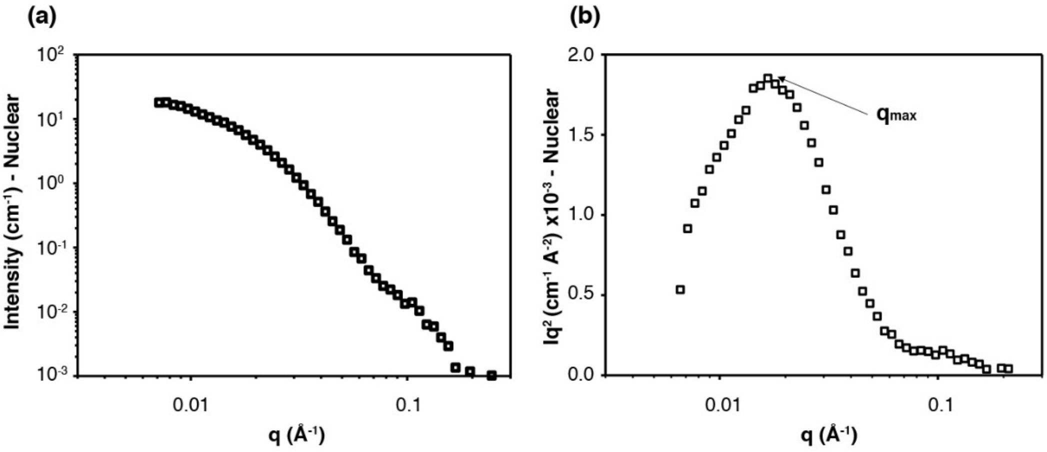

Supplementary Figure 11. SANS nuclear scattering data. a SANS nuclear scattering from the Stage 3 RAFM steel following subtraction of Porod Law behaviour and incoherent scattering background and **b** Kratky plot (Iq^2 versus q) of the Fig. 8 (a).
